# Jaborandi in the Treatment of Mumps

**Published:** 1879-09

**Authors:** 


### JABORANDI IN THE TREATMENT OF MUMPS.

Dr. Testa reports five cases of mumps treated with jaborandi. After free perspiration and salivation, there was a marked amelioration of the symptoms and the patients desired food. Next day the parotid swelling was much reduced, and in two days more the cure was complete. Dr. Testa thinks jaborandi acts by its hydragogue properties, that it may shorten the disease and prevent metastasis—*Jour. des. Sci. Med.*

